# Prevalent Morphometric Vertebral Fractures in Professional Male Rugby Players

**DOI:** 10.1371/journal.pone.0097427

**Published:** 2014-05-20

**Authors:** Karen Hind, Fraser Birrell, Belinda Beck

**Affiliations:** 1 Carnegie Research Institute, Leeds Metropolitan University, Leeds, West Yorkshire, United Kingdom; 2 Institute of Cellular Medicine, Newcastle University, Newcastle upon Tyne, United Kingdom; 3 School of Rehabilitation Sciences, Griffith University, Gold Coast, Australia; Faculté de médecine de Nantes, France

## Abstract

There is an ongoing concern about the risk of injury to the spine in professional rugby players. The objective of this study was to investigate the prevalence of vertebral fracture using vertebral fracture assessment (VFA) dual energy X-ray absorptiometry (DXA) imaging in professional male rugby players. Ninety five professional rugby league (n = 52) and union (n = 43) players (n = 95; age 25.9 (SD 4.3) years; BMI: 29.5 (SD 2.9) kg.m^2^) participated in the research. Each participant received one VFA, and one total body and lumbar spine DXA scan (GE Lunar iDXA). One hundred and twenty vertebral fractures were identified in over half of the sample by VFA. Seventy four were graded mild (grade 1), 40 moderate (grade 2) and 6 severe (grade 3). Multiple vertebral fractures (≥2) were found in 37 players (39%). There were no differences in prevalence between codes, or between forwards and backs (both 1.2 *v* 1.4; p>0.05). The most common sites of fracture were T8 (n = 23), T9 (n = 18) and T10 (n = 21). The mean (SD) lumbar spine bone mineral density Z-score was 2.7 (1.3) indicating high player bone mass in comparison with age- and sex-matched norms. We observed a high number of vertebral fractures using DXA VFA in professional rugby players of both codes. The incidence, aetiology and consequences of vertebral fractures in professional rugby players are unclear, and warrant timely, prospective investigation.

## Introduction

Rugby union and rugby league are intensely physical contact sports that place players at risk for frequent traumatic injury. This risk has increased in parallel with the introduction of professionalism to the sport, given that a greater advantage can be gained from larger, stronger players [24]. The changes in physiology and anthropometrics of professional rugby players, have led to a greater physicality in the game and an increase in the incidence of traumatic musculoskeletal injury of almost 20% since the 2009/2010 season [1], [16], [35]. Both codes of rugby involve significant contact and physicality, and the use of little or no body padding. The multiple physical collisions, tackles and high impact hit-ups repeatedly expose players to very high magnitudes of direct force [14]–[15], with reports of ‘g’ forces as high as 7–10 g during professional games of rugby [15].

Fractures occur when loads exceed the capacity of a bone to withstand them, and constitute up to 6% of all rugby injuries, although not frequently reported at the spine [2], [24]. In the general population, vertebral fractures (VFs) are under recognised and therefore under-reported [26], with only around 1 in 4 receiving clinical attention [6]. This lack of recognition is due both to the absence of notable symptoms, given that VFs commonly occur without pain of a sufficient magnitude to arouse concern, and there is often difficulty in determining the cause of symptoms [26]. This may be particularly the case for rugby players who, with a higher than average pain threshold and being accustomed to injury, may be less likely to report minor pain or symptoms. There is a high risk for symptomatic spinal injuries (including vertebral nerve root and disc injuries) in professional rugby union players; with incidence quantified at 10.9 per 1000 match hours [13]. However, the specific incidence or prevalence of spine fracture (asymptomatic or symptomatic) in professional players is not known. Although VFs are infrequently reported, all are associated with morbidity [39]. In professional rugby players, back injuries have the highest rate of recurrence of all injuries [33], and there is an increased risk of further fracture following an initial VF [39]. Vertebral fracture may also be a catalyst for degenerative disease, discopathy, kyphosis, back pain and neuroplaxia [27], [37].

Dual energy X-ray absorptiometry (DXA) has previously been used to assess and monitor body composition in professional rugby players [19]–[20]. Recent models of DXA now include a morphologic Vertebral Fracture Assessment (VFA) facility, which provides an accurate, valid and reliable method of identifying and grading VF, to a standard comparable to conventional radiography and accepted by the International Society for Clinical Densitometry (ISCD) [23], [36]. The densitometer acquires a high resolution radiographic image of almost the entire spine at a radiation dose of less than 1% of a comparable radiograph [23]. VFA is used clinically in patients with a high risk of fracture, and has been used in published research studies of VF in adolescents, and men and women aged 18 to 87 years [10], [23], [40].

Despite the known increased risk for back injuries in contact sports, no study to date has investigated the prevalence of vertebral fracture in a professional rugby or sports cohort. The objective of this study was to investigate the prevalence of VFs in professional male rugby union and rugby league players, through whole squad screening using DXA-VFA.

## Methods

### Ethics statement

Ethical approval for the study was granted by the Carnegie Faculty Research Ethics Committee (Leeds Metropolitan University, UK) in accordance with the Declaration of Helsinki, and all participants provided their signed, informed consent prior to taking part in the study.An observational study was conducted and all tests were performed at the same research centre, between November 2010 and May 2012. Ninety five UK-based male professional rugby league (n = 53) and rugby union (n = 42) players provided signed informed consent to participate in the study. There were no players who declined the invitation to participate in the study. Rugby league players were recruited from one Super League and one Championship team (all first team members), and rugby union players from one English Premiership club (all first team members). The minimum age for participation in the study was 20 years. All participants had been signed on professional rugby contracts for at least the preceding three years, and all were currently signed to contracts.

### Measurements

Participants wore light-weight clothing and removed shoes and any jewellery for all physical measurements. Standing height was measured using a stadiometer (SECA, Birmingham, UK) and recorded to the nearest millimetre. Body mass was measured with calibrated electronic scales (SECA, Birmingham, UK) and recorded in kilograms (kg) to the nearest 0.1 kg.

Vertebral fracture assessment (VFA) was performed in all participants, using a GE Lunar iDXA densitometer with the VFA software installed (Lunar iDXA fan beam densitometer with enCORE software version 13.5, GE Medical Systems, UK). The participant lay in a left lateral decubitus position according to the manufacturer's instructions for patient positioning, to ensure the spine was parallel to the table. The arm of the machine moved to the lateral position and then a lateral fan-beam high resolution X-ray image of the whole spine was obtained. Whilst spinal radiographs are generally considered to be the gold standard for the diagnosis of vertebral fractures [9], the VFA method is recognised as advantageous for being easy to use, precise and using low radiation [8]–[9], [23], . Furthermore, the iDXA VFA provides minimal interference from soft tissue artefacts around vertebral bodies, especially in the thoracic regions, whereas conventional spine radiographs require adjustment for soft tissue. The images acquired were of high quality and all vertebrae between L4 and T6 could be visualised clearly.

The automated quantitative morphometric analysis labelled vertebral deformations using a 6 point measurement of the anterior, posterior and mid points of the vertebras. In accord with the ISCD guidelines [36], each vertebra on all 95 scan images, were also visually inspected by a certified and VFA trained (ISCD) densitometrist, in order to minimise false positive or false negative results. Particular care was given to distinguish normal anatomic variants and non fracture abnormalities, notably Sheuermann's, degenerative disease, osteophytes, diffuse idiopathic skeletal hyperstosis, Pagets, Schmorl's nodes, cupid's bow defect, and rib and scapula shadows. Visualisation of vertebra was optimised using the ClearView facility which enhances images by adjusting contrast and brightness for bone edge enhancement. Once markers were agreed on, the software calculated the degree and type of vertebral shape anomalies using the semi-quantitative Genant classification [18], which is considered the most appropriate and standardised method [8]–[36]. Anomalies were classified as either wedge (when the anterior height was the lowest), biconcave (when the middle height was the lowest) or crush (when the posterior height was the lowest). A relative height reduction (with reference to posterior-mid-anterior heights) between 20–25% was graded ‘mild’, 26–40% ‘moderate’ and >40% ‘severe’. Precision for VFA by DXA has been reported at 0.84% CV for the average height of vertebrae in men aged 38 to 87 years [40].

Lumbar spine (L1-L4) areal bone mineral density (BMD) was evaluated using standard DXA procedures. Age- and sex-specific reference data was used to calculate BMD Z-scores. Body composition was measured in all participants also using DXA of the total body, and variables included fat percentage and lean mass. Local precision values for our Centre are 0.4% for lumbar spine BMD and 0.5–0.9% for body composition (in healthy subjects, aged 34.6 years) [21], [22]. The observed in-vitro coefficient of variation was low at less than 0.5% for the regular quality control scans of the Lunar (soft tissue and bone) calibration phantom. In this study, repeat scanning to perform a precision analysis of the vertebral deformity grading by VFA DXA was not feasible. However, published data demonstrates excellent agreement between DXA VFA and conventional radiography, with very good sensitivities and specificities, particularly for moderate (grade 2) and severe (grade 3) fractures [5], [23], [33].

### Statistical analysis

All statistical evaluations were performed using SPSS version 18.0 (LEAD Technologies Inc). Descriptive statistics (mean, standard deviation of the mean, minimum and maximum values) were used to characterise the sample. Data were normally distributed, therefore comparisons of descriptive results between groups (with VF *v* without VF; code: rugby union *v* rugby league; position: backs *v* forwards) were made using independent t-tests. Pearson's correlation analyses were computed to investigate relationships between dependent and independent variables. The level of significance for all tests was set at p<0.05.

## Results

Participant descriptive results are summarised in Table 1. There were no differences in age, BMI, body fat or BMD between rugby union and rugby league players (p>0.05). Rugby union players were taller (186.7 (6.9) *v* 182.3 (6.1) cm, p = 0.001), heavier (105.1 (12.9) *v* 96.1 (10.1) kg, p<0.001), and had greater lean mass (81.6 (7.6) *v* 75.4 (7.6) kg, p<0.001) than rugby league players. When participants were grouped by playing position (backs or forwards), there were 49 backs and 46 forwards. Forwards were taller, heavier, with a higher percentage body fat, lean mass, total body and lumbar spine BMD than backs (p<0.05).

**Table 1:** Descriptive results for the group of professional rugby players, n = 95.

	Mean ± SD	Minimum	Maximum
Age (years)	25.9±4.3	20.0	33.6
Height (cm)	184.3±6.8	167.0	202.0
Weight (kg)	100.1±12.3	69.0	137.7
BMI (kg.m^−2^)	29.5±2.9	23.3	38.5
% body fat	17.7±4.1	9.9	27.4
Lean mass (kg)	78.1±8.1	55.6	97.0
Total body BMD (g.cm^−2^)	1.33±0.11	1.33	1.87
Total body Z-score	3.6±1.2	1.1	6.7
Lumbar spine BMD (g.cm^−2^)	1.55±0.15	1.23	2.01
Lumbar spine Z-score	2.7±1.3	0.1	7.5

BMI: body mass index; BMD: bone mineral density.

**Figure 1:**
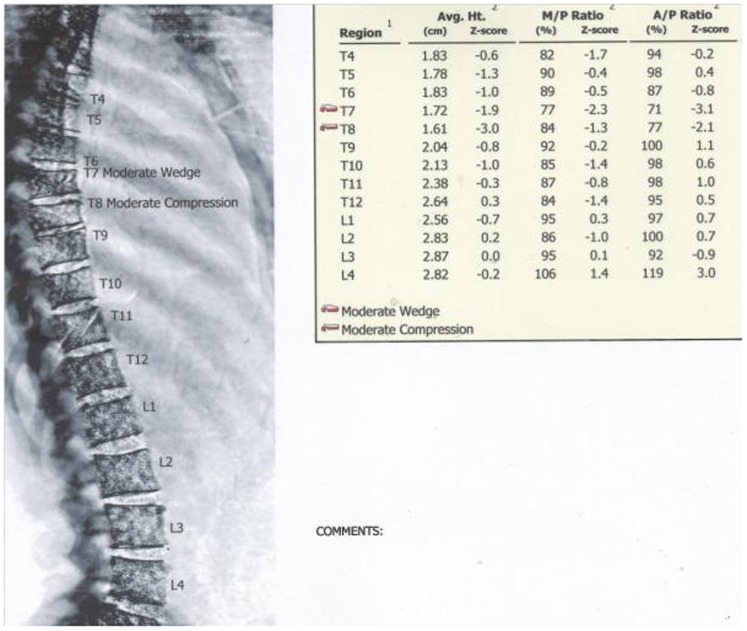
GE Lunar iDXA Vertebral Fracture Assessment report for a professional rugby league player, aged 28.1(T7) and one grade 2 compression fracture (T8).

**Figure 2:**
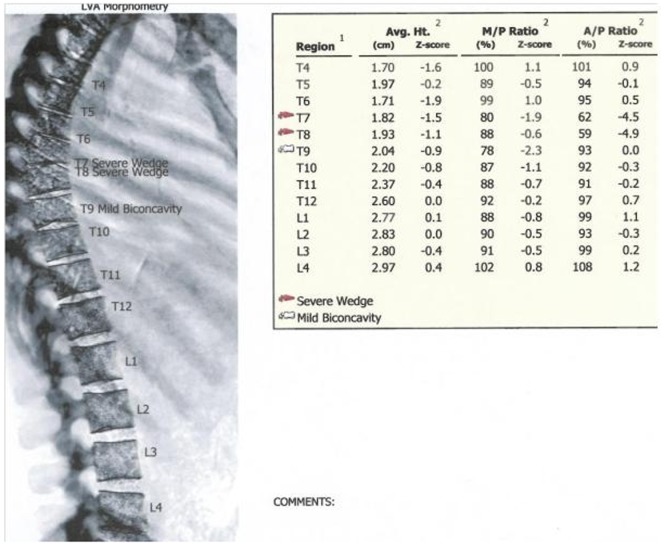
GE Lunar iDXA Vertebral Fracture Assessment report for a professional rugby league player, aged 23.6(T7 and T8) and a grade 1 biconcave deformity (T9).

**Figure 3:**
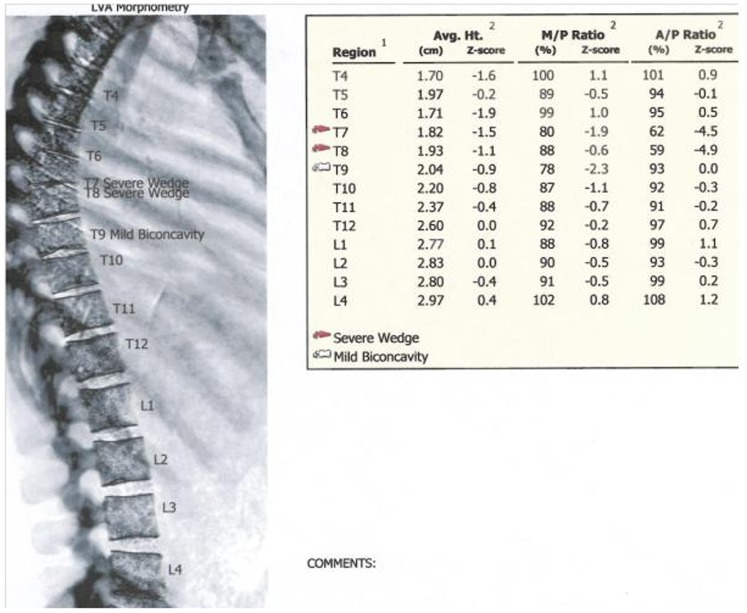
GE Lunar iDXA Vertebral Fracture Assessment report for a professional rugby union player, aged 31.3(T12), and three grade 1 wedge deformities (T10, T11, L1).

In total, VFA identified 120 morphometric VFs in 51 players. Examples of VFA images and morphometry from our sample are presented in Figures 1 to 3. Seventy four were graded as mild (grade 1) (62%), 40 as moderate (grade 2) (33%) and 6 as severe (grade 3) (5%). VFs by DXA were observed in 24 (57%) rugby union players and 27 (51%) rugby league players. Multiple fractures (>2) were identified in 37 players (39%). Moderate to severe VFs were identified in 31 players (33%). Three players (3%) were each found to have five VFs and each of these players had a deformity identified as ‘severe’. Five players (5%) had four VFs each (all with ‘moderate’, and two with ‘severe’). There were no differences in average number of VFs per player between rugby union and rugby league (n = 1.2 (1.4) *v* n = 1.4 (1.6); p = 0.731) players. There were no differences in the average number of VFs per player between forwards and backs (n = 1.2 (1.4) *v* n = 1.4 (1.6); p = 0.477). Furthermore, there were no differences in participant descriptive results between players with, and without VF.

The majority of the identified VFs were located in T8 (n = 23), T9 (n = 18) and T10 (n = 21). Fractures classified as severe (grade 3) were prevalent in T7, T8, T12, L3 and L4. The most common type identified was wedge (n = 50), followed by biconcave (n = 44) then compression (n = 26). All 6 severe VFs were wedge type. There were also no differences in the type or grade of fracture between rugby union and rugby league players. Bivariate correlation analyses revealed positive associations between lean mass and BMD at the total body (r = 0.462, p<0.001) and lumbar spine (r = 0.366, p<0.001). There were no associations between height and BMD. There were no associations between any independent variable and number, type or grade of VF.

## Discussion

This is the first study to investigate the prevalence of VF using DXA Vertebral Fracture Assessment imaging in professional rugby players and to examine differences in prevalence between rugby union and rugby league codes. The principal finding was that of a high prevalence of VF in a group of professional rugby league and rugby union players. The majority of VFs were found in the thoracic spine region, between T8 and T10. The rates of over 50% for all grades of fracture, and 33% for grades 2–3 (moderate - severe), are greater than prevalence rates cited for the general population [29].

There are few published reports of thoracic or lumbar spine fractures in rugby players. Most of the existing data are related to trauma to the cervical spine; likely due to the potentially devastating clinical consequences of injuries at this region [7]. The first report of thoracic spine fractures in a rugby league player was in 1997 [17]. Investigations were conducted after the player complained of persistent dorsal mid-thoracic pain, and revealed fracture to T6 and T7 without neurological complications [17]. Elsewhere, symptomatic spinal injuries have been reported in rugby union players, including nerve root injury [13]. Investigations in the current study were not based on symptoms, except for one case. One rugby league player (full-back) visited us with back pain the week following a significant collision with another player. He had received chiropractic treatment for his pain, which worsened and was assumed to be muscular. VFA revealed multiple clinical (with pain) fractures between T6 and T10 (severe at T8 and moderate at T9 and T10). The player rested but returned to play within one month.

Our findings suggest a higher risk for vertebral injury in players engaged in professional rugby of both codes, than previously recognised. There were no player characteristics that were predictive of number, type or grade of VF, and there were no differences in characteristics between players with and without VFs. It is common knowledge that a weak bone is less capable of withstanding force than a strong bone, and in the general population, fractures to the spine are most frequently associated with bone fragility [6]. Athletes from contact sports generally have superior BMD compared to non-athletes [11], supporting Frost's Mechanostat theory, which proposes that when all else is equal, individuals who load their skeletons to a high degree should have stronger bones than their less active peers [12]. The mean total body and lumbar spine BMD of our sample was well above age-matched normative values, and the lack of relationship between BMD and vertebral deformity suggests an underlying aetiology unrelated to bone fragility. Fracture risk is also dependent on the force of impact and the structure and geometric properties of bone [28]. The architecture of the vertebrae optimises strength and flexibility while minimising weight. It is not known if rugby player vertebral bone structure (such as bone size, cortical thickness, trabecular number, thickness, and connectivity) differs from the non-rugby playing population. Nor have the forces to which the spine is exposed during rugby playing been directly quantified. As a consequence, the magnitude of force to the spine in rugby that will exceed the threshold of vertebral bone tolerance is unclear. The prevalence of the observed VFs in rugby players however, may indicate that peak strain magnitudes in the spine generated during tackling and in collisions, or significant hyperextension, can exceed the vertebral bone strain threshold [12].

It has been estimated that more than two thirds of a ton in force is shared across the front row of players during the engagement of the scrum in rugby union and that this is associated with a greater risk for spinal cord injury [4], [31] prompting calls for a ban of the contested scrum [3]. Our finding of a similar prevalence of VFs in rugby league and rugby union players, despite the absence of the contested scrum in rugby league, suggests additional causes of VFs may also be at play. Indeed it has been suggested that collisions have the highest propensity to cause traumatic injury during rugby [13], [15]. Collisions are not only limited to game situations - an average of 77 collisions per player have been recorded during National Rugby League team training sessions [15]. The demands on professional players to maintain training and game time may increase vertebral micro-damage and muscle fatigue thereby exposing the spine to even greater mechanical stress during impacts. Micro-damage to bone tissue that occurs in the course of regular mechanical loading is repaired through a process of bone remodelling so that overall bone strength is maintained. When the rate of micro-damage accumulation is greater than the rate of repair, bone is more susceptible to structural failure. Medical management of pain in professional rugby players, including the use of steroid injections, local anaesthetic and chiropractor therapy [34] may exacerbate vertebral bone injury. These interventions may also return players to the game before micro-damage can be repaired, thereby exposing them to increased risk of fatigue fracture at the spine. Research is required into both the direct and indirect localised effects of analgesic interventions on bone in elite sports populations.

The long term consequences of VF in professional athletes from contact sports, is unknown. A recent study reported an 11% increased risk of mortality among Olympic athletes from contact sports compared with other athletes [41]. The authors suggest their finding reflects the impact of repeated collisions over time and hypothesised that the risk may be underestimated for the current generation [41]. Of further clinical relevance, peak bone mass can continue to accrue in the third decade of life, therefore the skeletons of many of the current study participants may not have stopped growing (mean age 25.9 years). The effects of repetitive high trauma to the growing spine are unclear.

Our sample was restricted to professional rugby players participating at the highest standard, and at this level, the intensity of collisions and other physical contact is likely to be considerably greater than at the amateur level. Our results are therefore not to be generalised to all rugby players. Although we did not test a control group, epidemiological data elsewhere has reported 1.0 to 4.0 incident VF cases per 10,000 population/year in men, 20–35 years of age [38]. It was also not feasible to conduct spinal radiographs, however, the VFA method is widely recognised as advantageous for being accurate and precise, mostly for grade 2 and grade 3 vertebral fractures [5], [23]. There are no sensitivity data specific to young adult male athletes, however precision of 0.84% in men aged 38–87 years for VFA has been reported [40], and VFA has also been used in younger age groups with success [10]. Due to the observational design of this study, we cannot assume cause and effect, or rule out other potential causes of VF linked to general risky behaviour. However, an increased risk for musculoskeletal trauma is inherent to contact sports. The health consequences of repetitive physical trauma, during muscular fatigue and over time, in professional rugby players remain unclear. Chronic back pain is one of the most common complaints of retired rugby players [30], although back pain is not always attributable to VF [25]. Further studies should be prospective in design, and would benefit from the inclusion of an assessment of back pain scores in players.

In conclusion, this study is the first to investigate and reveal a high number of vertebral fractures in professional male rugby union and rugby league players. This identified risk warrants exploration in prospective studies in order to quantify seasonal incidence, relative risk, and investigate possible aetiologies. We recommend that pre and post season vertebral fracture screening protocols for all professional players, are considered by rugby league and rugby union governing bodies and clubs. We also recommend the development of sport-specific vertebral fracture safe-management guidelines. Finally, the effects of contact sports on the development of the growing spine, and the short and long term consequences of VFs in professional rugby players, are unknown and represent additional avenues for timely, future research.
